# Nurses Lived Experience of Transition From Bedside Nurse to Nurse Manager in Jordan: A Qualitative Study

**DOI:** 10.1155/jonm/1072596

**Published:** 2026-02-10

**Authors:** Raya Yousef Alhusban, Omar Mansur Alshareet

**Affiliations:** ^1^ Faculty of Nursing, Zarqa University, Zarqa, Jordan, jadara.edu.jo; ^2^ King Hussein Cancer Center, Amman, Jordan, khcc.jo

**Keywords:** Jordan, nurse, nurse manager, nurses lived experience

## Abstract

The transition from bedside nurse to nurse manager can be challenging, and it needs more studies in Jordan. Therefore, this qualitative study aimed to explore the transition experience from a bedside nurse to a nurse manager in a governmental hospital in Jordan. The researcher utilized an interpretive phenomenological approach. Data collection included audio‐recorded face‐to‐face semistructured interviews with 12 full‐time nurse managers. The findings of this study identified six themes representing the experience of transition from bedside nurse to nurse manager: (1) swapping their nursing uniforms for administrative suits seeking transformation, (a) guidance and support: hand in hand and (b) nurse manager is a servant leader; (2) struggled to make a difference while being pulled in all directions with subthemes (a) single‐handed, (b) accountability without authority; and (3) sewing the executive suits with empowerment. In conclusion, the findings of this study indicated that the experience of transition from bedside nurse to nurse manager includes positive and negative aspects that are manifested by a willingness to wear the administrative suits, support, and guidance received either from the manager or from the staff nurses, and the presence of networking and empowerment to be effective as a new nurse manager. At the same time, the negative experiences were a lack of preparedness, lack of autonomy, frustration, and meeting the needs of top and lower managerial levels. Healthcare administrators must implement specialized training programs and assign mentors to facilitate the transition from bedside nurse to nurse manager, ensuring that new nurse managers are qualified to improve patient outcomes.

## 1. Introduction

Healthcare is a challenging work with complex requirements. Nurse managers (NMs) are essential for achieving organizational objectives and missions and establishing secure, healthy workplaces that facilitate the work of the healthcare team and improve patient outcomes. Around 1.1 million additional registered nurses are required to satisfy the growing demand and replace retiring workers, many of whom hold managerial positions [1]. The need for medical and health service managers is expected to increase by 28% between 2021 and 2031, which is substantially faster than the average for all occupations [1]. A NM is a registered nurse who may be directly or indirectly responsible for supporting and coordinating nursing staff and delegating tasks to achieve the health organization’s goals [2]. A NM works as a head nurse, unit manager, ward manager, or ward head nurse [3]. NM roles include directing nursing care in organizations that provide inpatient clinical nursing care. The role includes hiring, supervising, and evaluating nursing staff and managing payroll and budgets [4].

Nursing professionals seeking to transition from bedside care to managerial positions must possess strong leadership abilities. More specifically, Gaalan et al. [5] expect them to possess skills in financial management, negotiation, team recruitment and development, conflict resolution, and technological advancements, Gaalan et al. [5]. A nursing manager must have rational thinking and skills to achieve the healthcare organization’s goals. Additionally, they should be a problem solver and work under pressure; they must also have the necessary education, training, and emotional intelligence to control their emotions [6]. Additionally, a trained manager can stabilize workload and stress and boost productivity at work to enhance the delivery of high‐quality care to patients [7]. Typically, modeling and competency‐based learning develop managerial skills [7]. Although nursing shortages continue, the number of nurses who choose to enter management may decline [1].

Today, the managerial role of nurses depends on rapid technological changes, communication style, information transparency, patient needs, service quality, and compliance with rules and standards [8]. Role requirements for NMs in hospitals have become extremely complex and challenging due to the complexity of the healthcare delivery system [3]. NMs occupy various positions in their daily routine. So, some adopt an effective leadership style, while others may have difficulty in assuming the managerial role [3]. The workplace is characterized by challenges and constant change, and most NMs are tasked with managing a large number of staff and patients [9]. Consequently, many nurses may leave their managerial role to return to staff nursing work or withdraw from the nursing profession altogether [10], and about 30% of NMs intend to resign which can adversely impact patient outcomes [11].

One of the most important aspects of nursing management is finding nurses who can occupy key NM position. As a proactive method of identifying and developing future NMs, the organization’s strategy should include strategic NM succession planning and smooth transition. Doria [12] noted that because NM must complete years of training before being fully competent in their fields, their loss could result in declining institutions’ functioning and financial losses [13]. According to Ga Allah [14], NMs are typically chosen based on clinical skills and often lack the professional education and support needed for effective NM transfers. Other causes of the transition process’s weakness include the unwillingness of the old managers to share their expertise and experiences with possible managers [13]. Another hardship that may decrease the competence among NMs is the lack of experience, which may significantly impact how well they execute in management roles [15]. A cross‐sectional study aimed to identify techniques for a successful transition to NM explored four main themes: mentorship, coaching, knowledge, and development programs [9].

Another finding showed that, to handle complex problems, NMs must also be accessible around‐the‐clock, without the necessary equipment, resources, and structures to support their demanding job, which might dramatically increase their stress levels, causing them to get exhausted, and rendering them incapable of carrying out their expected duties [3]. Investigating staff nurses’ lived experiences as they transition to the NM role revealed a lack of leadership and support [16]. Besides, many NMs lack the education and support they need, and as a result, they lack the tools, infrastructure, and resources to perform their new administrative function [3, 16]. Furthermore, inadequate role preparation increases the risk of role confusion and role stress, which decrease the effectiveness of the organization and can lead to a high attrition rate [3]. Another problem is the difficulty of retaining the current NMs, due to the ambiguity of job functions and the high‐stress levels associated with unrealistic expectations related to achieving goals [14]. Perceptions that management roles are not influential in inducing change and unpreparedness for manager roles both hinder the transition of nurses into managerial roles [16]. Exploring the experience of nursing managers is critical to avoid negative impacts on nurses’ satisfaction, healthcare costs, and patient outcomes [17]. In Jordan, it is essential to understand NMs’ lived experience of transition from bedside to an NM role so that they may jointly maximize role efficiency and effectiveness [3]. As well as, in Jordan, there is a scarcity of studies that explored the manager’s lived experience in the context of public hospitals, and there is a need to pinpoint the difficulties and barriers that the NM faced during transition and work to overcome them. Additionally, this study can help the Jordanian Nursing Council (JNC) ensure a qualified nursing workforce capable of leading and managing complex social, economic, and political challenges, as well as actively influencing healthcare systems for better outcomes. Therefore, the main aim of this study is to understand the NMs’ lived experiences among Jordanian nurses. More specifically, this study’s purposes include the following:

1. Identifying the difficulties encountered by the NM while transitioning from a bedside nurse to a NM role.
2. Exploring the facilitating factors that help in the smooth transition from bedside nurse to NM.

## 2. Methods

### 2.1. Design

Researchers used an interpretive qualitative phenomenological research design to elucidate the whys and hows of the transition from a bedside nurse to an NM. Phenomenology is a method and philosophical inquiry developed by Husserl and Heidegger [18]. Researchers chose phenomenology for this study due to its ability to provide a more profound understanding of the self‐involvement experience of NMs within the context of Jordanian healthcare. They derived the structure of the lived experience description from an objective analysis of interviews, transcripts, field notes, and reflective writing. Furthermore, in the process of interpretive inquiry, through interaction and interpretation, the participants and the researcher develop a common understanding of the fusion experience. The researchers used interpretive phenomenology in this study to investigate how subjects make sense of their social and personal environments and give specific experiences, feelings, and meaning [19] (p. 33–34). Interpretive phenomenological analysis (IPA) provides detailed examinations of personal lived experience. Hermeneutics, also known as “interpretive phenomenology,” is used to describe, understand, and interpret participants’ experiences [20] (p. 41–42).

### 2.2. Settings

The participants of this study were recruited from a large public referral hospital in Amman City. It has around 75 NMs and head nurses. It can accommodate 1100 beds currently, and it includes 49 buildings and 80 departments under the management of the Jordanian Ministry of Health [21]. All regions of the Kingdom refer patients to this transformative hospital [21].

### 2.3. Participants

The researcher used purposeful sampling of 12 NMs. The inclusion criteria for this study included NMs at all levels of management except the nurse director, both genders, and those working in all nursing departments, holding at least a bachelor’s degree in nursing, with at least 6 months of experience as a current nursing manager who had/has a full‐time job in this role. The exclusion criteria were NMs with less than 6 months of experience because they will not enrich the study due to the short time in the position as manager, and diploma nurses, since they will not be hired as NMs. The initial sample size was determined based on data saturation [22].

### 2.4. Data Collection Plan

The researcher approached the predetermined hospital and got permission from the directorate to access the NMs in the selected departments. Then, the researcher invited the NM to enroll in the study after screening them according to the inclusion criteria. The principal investigator approached the possible participants in person to fully explain the purpose and method of the study and the roles and responsibilities of the participants. After they agreed to participate and then signed the process consent form, the researcher asked the participants to complete the demographic data sheet. The researcher used a semistructured face‐to‐face interview with a predetermined interview guide developed based on the related literature. The researcher conducted the 30‐ to 45‐min interview in NM’s office, a private location within the hospital. The researcher obtained the participants’ permission to voice‐record the interview. The researcher recorded the participants’ nonverbal communication during the interview and supplemented it with notes. Thereafter, researchers analyzed the data in Arabic, and the results were transcribed verbatim into English.

### 2.5. Ethical Consideration

The human rights of the participants were assured by initially obtaining approvals to conduct this study from the ethical committee at the selected university’s Faculty of Nursing (No1/2022). Then, approval was granted from the ethical committee of the Ministry of Health (No. MOH/REC/2022/376). The researcher then told the participants they could leave the study at any time. The researcher maintained confidentiality of the data by keeping all the data in a password‐protected computer that can only be accessed by the researcher, besides maintaining anonymity throughout the study by using numbers.

### 2.6. Data Analysis

The researcher used IPA, which has descriptive and interpretive components and enables intersubjective understanding [23]. After transcribing the data, the researcher works closely and intensively with the text, annotating it carefully (‘coding’) for insights into the participants’ experiences and perspectives on their world. The steps involved in the IPA process are as follows:

1. Individual transcripts are read, and significant phrases are extracted. The researcher catalogs the emerging codes and subsequently begins to look for patterns in them. These patterns are called “themes.”
2. The researcher identifies emerging themes from these phrases.
3. Themes from all transcripts are collected together, and possible connections between themes are noted.

The researcher then examines the original transcripts to determine if participants’ actual discourse provides sufficient evidence to support the themes. The sixth phase is creating structural descriptions of participants’ overall experiences, which are based on the textual description that was previously provided.

### 2.7. Trustworthiness of the Data

In this study, the researcher addressed the trustworthiness of the research findings through credibility, transferability, conformability, and dependability [24]. Credibility was achieved by prolonged engagement with the participants and the context of the study and by member checking, whereby the study findings were discussed with a sample of the participants after taking their permission. Then, the researcher double‐checked the transcripts and data analysis to enhance the reliability of the findings. Thereafter, the researcher will use thick and rich descriptions supported by participants’ narratives to enhance transferability. The researcher achieved conformability by consulting an external auditor who is an expert in qualitative research to review the whole process of the study steps.

## 3. Results

A total of 12 participants completed the semistructured interviews. The mean age of the participants was 33 (SD = 8.67). Most of the study’s participants were males and had bachelor’s degrees in nursing. The majority of them had an average of more than 15 years of clinical experience. The sample’s essential demographic characteristics are listed in Table 1.

**Table 1:** Sample characteristics (*N* = 12).

Variable	Category	*n*	Percentage (%)
Gender	Male	8	66.66
	Female	4	33.34

Age	22–28	1	8.34
	36–42	8	66.66
	43–49	3	25.00

Marital status	Single	1	8.33
	Married	9	75.00
	Divorced	2	16.67

Monthly income	> 500 JD	9	75.00
	500–1000 JD	3	25.00

Experience in nursing	0–9 years	1	8.33
	9–15 years	3	25.00
	< 15 years	8	66.67

Education level	Bachelor	10	83.33
	Master	2	16.67

Years in manager role	0–3 years	4	33.34
	3–9 years	8	66.66

### 3.1. Findings of the Study

According to the study’s findings, the transfer of a Jordanian NM into the position of NM was both a positive and a negative experience. Five themes emerged: (1) swapping their nursing uniforms for administrative suits seeking transformation, (a) guidance and support: hand in hand and (b) a NM is a servant leader. (2) Struggled to make a difference while being pulled in all directions with subthemes: (a) single‐handed, (b) accountability without authority, and (c) sewing the executive suits with empowerment, as illustrated in Table 2.

**Table 2:** Themes and subthemes of the study.

Main theme	Subthemes
1. Swapping their nursing uniforms for administrative suits seeking transformation.	a. Guidance and support: hand in hand
	b. Nurse manager is a servant leader

2. Struggled to make a difference while being pulled in all directions with subthemes.	a. Single‐handed
	b. Accountability without authority
	c. Sewing the executive suits with empowerment

#### 3.1.1. Theme One: Swapping Their Nursing Uniforms for Administrative Suits Seeking Transformation

Participants described their bravery and firmness in swapping their nursing uniforms for administrative suits while transitioning from nurse to NM. At the same time, they reported their feeling of unsatisfaction at times, when they felt upset and unprepared for the manager role. Furthermore, participants were uncertain between leaving their adored patient care role and taking on a new role that could be “fruitful,” as one of the participants reported:

*“For me, it was, ‘here’s your office, figure it out,’ and that’s what I’ve been doing. One of the hardest things, in the beginning, was that I’m a bedside nurse, and that’s where my heart is because, at heart, I’m a nurse. You have to start thinking somewhat differently. So I had to learn to balance the two, the nurse and the manager.” Participant 5*

On the other hand, some participants were optimistic about wearing the new administrative suits to advance in their careers because they wanted to change their bedside nursing role to a managerial one. As a result, they were eager to take on the new administrative role, as Participant Number 11 reported. “*I was pleased to be promoted to management, and I hope to be capable of handling this responsibility.”*

Participants reported wearing the administrative suit either because some managers and colleagues believed in their management abilities or because the previous manager was leaving, creating an opportunity to fill the position [25].

As stated below:

*“I think someone saw that I had potential and taught me things. One day my former boss called me and said, “Hey, I was just wondering, and I’m not sure what you want to do, but there will be this opening, and I think you will be suitable for the manager role.”* Participant 3

Participants reported their desire to change their bedside suits to NM ones, and with the colleague’s encouragement and support, they accepted wearing the administrative suits, and it could be an “opportunity” to advance their careers through the role of nursing manager.

*“It was an opportunity … I wanted to know if it could be a good fit; I was trying to help to change my job title and my satisfaction for myself and my employees. It could be a springboard for my career. I didn’t want to remain in a bedside role for too long; I aimed to transition into management, which I believe is a good starting point.” Participant 1*

*“I was in the right place at the right time; now the previous NM decided to step up and quit.” Participant 6*

Many participants stated that their primary motivation for applying for a management position was positive relationships with people who had mentored or influenced them. Participants also hoped to foster camaraderie among nursing staff in order to “bring the units together” through business relationships.

“*I didn’t really know I wanted to be a manager, and so the former director [deleted] encouraged me and said, ‘You know, you’re a natural at the NM role; you can do this, you can do this.’ All my fears and questions to my supervisor were answered, and then she (the supervisor) persuaded and reassured me; she was just like a wonderful teacher.”* Participant 8

##### 3.1.1.1. Subtheme (a): Guidance and Support: Hand in Hand

Participants reported that, with the help and guidance of supervisors and colleagues, they are attempting to participate actively in their new NM position. Despite their burden and feeling unprepared for the NM role, participants received direction and care from colleagues. They described how colleagues encouraged them to maintain their bravery and directed them through the complex world of management, allowing them to become confident and successful [25].

“*I worked with the person who was the director. She had really guided me in many administrative matters. So, when [name omitted] left, the next natural thing was for me to take over the NM role*.” Participant 1

*“I was very hesitant at first, but I kept stressing that it would be a temporary role, and that’s what I kept focused on. So, after that temporary period, the moderator said, “You know, you’re doing a really good job. I really think that you should take this stand.” Participant 10.*

Through open and honest communication, participants were rewarded for their willingness and commitment to the hospital; they were influenced by their supervisor and colleague to pursue the role of an NM, and they believed they would support them during their transition.

*“If you are encouraged and supported, and they like you, you stay there or advance to the next management role. I think it was people who guided me and people who believed in me. If you had someone who believed in you and wanted to guide you.” You can do it and move on. I think empowerment comes from those you talk to or who have guided you.” Participant 7*

*“They didn’t feel like there was anyone else capable of managing it. I tasted it a bit and realized I was really capable. I felt like in that moment people came and went, and they couldn’t make it work. And I just thought, ‘You know what, I think I can do this.” Participant 11*

##### 3.1.1.2. Subtheme (b): NM is a Servant Leader

Participants believed that nursing was about helping others, acting as their manager, and resolving subordinate issues while leading them through service. Consequently, they characterized the role of an NM as embodying a servant leadership style. According to one of the participants, focusing on servant leadership entails giving without expecting anything in return [25], as stated by one of the participants.

*“I believe you must listen to your employees, including their wants and needs. This approach is similar to how you would listen to your patients”. Participant 1.*

Participants stated that “NMs are servant leaders, which means guiding, coaching, and being part of the team.” Participant 3: “Another participant perceived the NM role as servant leaders who entered nursing to help others, either their patients or their colleagues and subordinates.” When asked about their service leadership practices, participants stated that they go beyond their office and look for the needs of their subordinates by “guiding” them, which “builds trust.” Participants explained how the concept of servant leadership led them to want to guide and nurture others; it was clear that active listening and a hands‐on approach were important aspects of servant leadership.

Participants reported that they did not receive courses or training workshops about the style of leadership; rather, they were nurtured to take care of others, while some participants acquired the knowledge from their postgraduate academic education.

*“I always talk to them, and guide them, and listen to them. So, I surface my problem so I can help them?” Participant 4*

*“They know I’m always there for them if they have a problem, they’ve had a difficult day, and they need someone to help them deal with situations.” Participant 6*

#### 3.1.2. The Second Theme: Struggled to Make a Difference While Being Pulled in all Directions

The participant chose to become a NM because they were enthusiastic about improving the unit’s conditions. They expressed a desire to improve both patient care, the patient’s environment, staff capabilities, and their working conditions. They appreciated the autonomy of the new role, which allowed them to have a greater impact on their nursing practice and the staff as an NM rather than a bedside nurse.

“I had good ideas, I know everything in my department, and I can fix many things with the help of employees and the nurse manager’s assigned responsibilities.” Continue… Participant #5

Participants discussed how working as a staff nurse with patients would increase their efficiency as NMs in implementing changes to improve patient care. Furthermore, participants reported stagnation in their bedside jobs, prompting them to decide to change their roles, as exemplified by Participant Number 10.

The participant showed a lot of empathy and a desire to help others based on their own experiences with staffing problems and problems at work. They thought that their firsthand knowledge of these issues put them in a good position to help, as they believed they could help meet the needs of their department and make a real difference.

Those who trusted participants urged them to take the NM role and improve their careers, as one participant stated:

*“For example, Things that were well established and taking for granting for many years without change, that I was disagreed maybe I can help change them.” Participant 3*

*“If you are a manager, you can participate in higher-level administration and attend those meetings.” I want to be more elaborator in decision-making and that kind of thing.” Participant 6*

Participants described the importance of guidance and support provided by their colleague; however, they were suffering as a manager to meet the needs of their headmaster and their subordinate while upgrading the quality of care provided to their patients. As they described, they were pulling in all directions, as stated: *“I am trying to keep as calm and meaningful as the budget fits, but still keep employees happy.”*

Participants perceived that being a manager was a great chance to help and create positive changes to patients, nurses, and the organizations. However, they were astonished to serve the upper and lower levels of management with great emotional costs that resulted from conflicts at all levels *“Many of these people continue to believe they are against me, which is emotionally difficult for me. Initially, I cried every day. Sometimes you feel alone.” Participant #2.*

##### 3.1.2.1. Subtheme (a): Single Handed

One of the big challenges that the participants face is lack of preparedness to be a manager when they are transitioning into the new NM role. Participants explained that unprepared new NMs board on an unplanned administrative journey without the resources and support to guide them.

“*Unprepared new nursing managers embark on an unplanned management journey without the resources and support to guide them*.” Participant 3

Participants in this study rejected Statement A, citing the fact that nurses typically have inherited management characteristics. However, they expressed concern that staff nurses may lack the necessary skills to effectively transition into the role of NM. As a result, some participants perceived insufficient potential among staff nurses to assume NM responsibilities, as well as inconsistent and ineffective approaches to preparing nurses for management positions.

*“The old NM didn’t make any kind of promises. He said, ‘I don’t want to tell you the map… You must find your own path. Occasionally they set you up for failure.” Participant 4*

Participants felt unprepared for their new NM role, and they were unfulfilled. In addition, participants were happy with the new chances they had in the NM role, but they also described the hindrance of transitioning into it without support and preparation. “*I ended up getting the position, but I’m totally unprepared for it*.” Participant 11.

Another challenge faced by the new NM was relationship conflicts. The relationship conflict leads the NMs to have emotional stress that affects them personally and forces them to change their behavior.

They said, “You are so different from me now,” or “This job has augmented me mentally.” Adding the responsibility of the NM role led the NM to feel “too stressed” due to interpersonal conflicts and high workload, and they did not receive help, as stated by Participant Number 12.

*“Strained relationships with senior management were evident as participants shared, ‘There’s not much direct control’ or ‘I couldn’t control or reform,’ which limited their passion to make changes, and I have high expectations, and work has become more difficult.”* Participant 1

*“Every day you come here (hospital)—there’s no such thing as [a] normal day. Its fire drills, bells and whistles, and sirens, and I think we’re all struggling.”* Participant 7

##### 3.1.2.2. Subtheme (b): Accountability Without Authority

Participants reported living difficult times, hardships that consumed every moment of their time. Some participants reported frustration due to the lack of autonomy that prevented them from making the desired changes and questioned their decision to remain in the role of NM [25]. Participants who also contradict the expectation of the subordinate, as illustrated by one of the participants:

*“I had some situations where I was uncomfortable with management decisions that were made that I didn’t agree with. That was something I really struggled with.”* Participant 5

Hindrance with the situation and uselessness in satisfying employees and senior management needs caused NM to question their tenure. Some NMs explained that they would “leave a few times,” as there was a limit to how much more they could earn. Some NMs reported “politics,” “a conflict of values,” or “my heart just wasn’t in it” as turning points in their decision to leave the NM role. One participant said that when she was provided the opportunity to leave, she felt “a weight lift” because she had moved from the position of NM to the bedside nurse.

“*When I leave the manager role because you feel worthless, I feel as if I were beaten because I looked like I was beaten and I wasn’t making anyone happy.”* Participant 4

*“Mole game at the fair when you flop your head up and get up, hit it with a rubber mallet: how many times will you raise your head?”* Participant 4

Participants feel committed to their subordinate, but their feeling of frustration due to the lack of autonomy forced them to plan to take off the administrative suits and leave the administrative position, as illustrated below:

*“I feel like I’m making a difference, and I feel people depend on me, and I would rather not be away from them, but I’m not sure how long it will take me to be here. To be completely honest, if the right job comes along, I probably won’t stay. And I don’t know if I’ll stay.”* Participant 6

##### 3.1.2.3. Subtheme Three (c): Sewing the Administrative Suits With Empowerment

Participants provided suggestions such as coaching for extended period of time to prepare new nurses for an NM role because the process of selecting a suitable candidate often goes unnoticed or neglected, according to participants.

Altogether, they also realized that the new nursing manager lacked the support and resources available from knowledgeable and experienced managers who could not only share skills but also offer reassurance and encouragement. Participants reported a lack of clear policy about the transition from bedside nurse to NM, such as training and education. Participants described how the perception of nurses’ appropriate capabilities automatically determines their suitability for a career in nursing administration.

*“I think support and information are the most important things.”* Participant 2

*“Nursing management training should be more focused on people skills rather than budgeting, buying, and selling.”* Participant 6

They explained that being a good skillful nurse does not guarantee great leaders, and some hospitals have limited areas for nurses to expand their managerial skills. So NMs advance into managerial positions due to the inspiration of their supervisors and nursing staff or under “pressure” without being properly assessed.

Participants suggested that assessments of “strength,” “confidence, and delegating responsibilities” are important to the success of their job before moving a nurse into a manager role. Evaluating potential candidates for managerial roles is important. However, participants also identified the need for new NMs to have preceptors who would support them and “know whom to ask,” as well as “weekly meetings” creating an avenue for encouragement and guidance.

The participants mostly agreed on the most important skills that a new NM needs to have in order to do the job well. They stressed how important it is for NMs to have a good “business sense,” which means knowing how to budget and buy supplies. Participants also stressed the importance of giving people power through training in interpersonal skills such as how to talk to people, how to handle conflicts, and how to discipline people, since these topics are often left out of traditional nursing education.

*“Maybe the nursing manager’s guidance program needs to develop a better nurse manager”. I don’t think we are doing an impressive job of preparing and training them for their roles.”* Participant 11

*“We tend to assume that because you’re a good nurse, you can do all these things—manage the budget, source staff, counsel staff, and evaluate staff … and we don’t.”* Participant 5

## 4. Discussion

To start with wearing the administrative suits to be a NM, most of the participants in this study reported a lack of readiness for the role of manager, and they were appointed due to opportunity, networking, or the presence of some leadership characteristics. This study is similar to the following studies [26]; Hanse et al., [27]. In addition, most of the participants’ decisions were based on their relationships with those who directed or inspired them to consider management roles. Fleiszer et al. [28] noted that one of the ways to manage is through the influence of others. The study findings also supported the initial conception of structural empowerment, which consisted of open communication, available resources with contacts, and support, including professional development opportunities. The study by Boamah et al. [26] focused on senior management, management approaches, and empowerment of nursing managers. The authors proposed that individuals feel appreciated when they are acknowledged for their contributions.

Participants in this study eventually questioned their initial decisions to change their bedside nursing career to an administrative one based on the belief that they could make things better from an unfulfilled managerial position. Kretzschmer et al. (2017) stated that “Accountability without authority—responsibility for outcomes without access to resources—creates frustration and failure.”

The participants in this study reported that their motivation to become managers was driven by a desire to change their professional status. This is in line with Wong et al. [26], who focused on nurses’ aspirations for management. Their research revealed that less than 20% of those who participated (*n* = 125) would choose to be a manager. The results indicated that role perception was an important factor in deterring nurses from a career in management. The participants in Boamah et al.’s study [26] agreed that there is a need for strategies that organizations can implement to help nursing staff develop as prospective managers.

The theme of Sewing the Administrative Suits with Empowerment indicated that participants cited a lack of support, ambiguity regarding their formal authority, and a lack of autonomy as factors that caused them to be trapped with multiple tasks and unrealistic expectations that made them question whether to remain in the Nursing Director position or not. Quinn et al. [29] showed that managers can create change by inspiring and empowering others. Participants in this study expressed the importance of independence in decision‐making, as they believed they would be able to influence and create positive change through their contributions to their units in the role of NMs. Boamah et al.’s [26] study supports these findings, with individuals reporting increased levels of commitment when they relate to and are part of the change. Furthermore, these findings support the three components of the empowerment framework, in which there is a link between increased psychological empowerment, structural support, and the experience of achievement in one’s job [30]. Participants experienced frustration as the expectations of others overshadowed their perceptions of their ability to positively influence. The findings of this study are in agreement with Havaei, Dahinten, and MacPhee [31], who indicated that new NMs were less likely to stick to the organization rules if there was an expectation to manage a large number of staff with limited support. In addition, McPhee, Skelton Green, Bouthillette, and Suryaprakash [30] identified management coaching as a way to empower managers with tools to use in their daily practices.

Participants in this study perceived the NM as a servant leader, and the main passion to stay in the managerial position was to serve others. The servant leader has the following behavioral characteristics: listening, empathy, healing, perceiving, persuading, envisioning, foresight, overseeing, and commitment to the growth of others and community [27]. Participants provided their experiences of practicing these characteristics of servant leadership. Managers who display and experience the characteristics of servant leadership have greater success in motivating and enabling their employees to participate and be committed to their organization Boamah et al. [26]and Hudgins et al. [32] also identified the necessity of recognizing the managerial potential of junior nurses due to the expected lack of turnover for both nursing managers and nurses. The NMs of tomorrow may not be ready, but with specific training to empower them, they in turn will enable future generations of staff nurses to develop into NMs .

### 4.1. Implications for Nursing Practice and Administration

This study offers information about the transition from a bedside nurse to NM, which has important implications for enhancing the knowledge and skills of new NMs and increasing the retention of managers in the role. Each hospital can establish a program that might positively enhance the skills and practices of NMs, called the new leader preparation program. Also, the hospital administrator is required to implement plans of trainers for trainees in management roles to create specific training programs for nurses and nursing managers. Hospital administrators have also bolstered their support for the new NM, granting them greater freedom to take action. In addition, effective communication and collaboration are critical for any team to function well.

Investing in NM leadership skills and training would benefit the organization financially through employee retention, particularly of experienced staff, as the nursing shortage continues to grow. The following recommendations are needed to ease the transition from the staff nurse to the NM role: (1) integrate the leadership and management program into the nursing curriculum at all levels from bachelor’s degree to doctoral degree, (2) provide a continuing professional program with a focus on the applied training courses to improve knowledge and skills about the NM role, (3) establish a mentor role to support the new manager role’s abilities and capabilities, (4) hospital administrators should empower new nurses and give them autonomy in decision‐making, (5) using the servant leadership model as an especially effective way to motivate the new NM, (6) open and honest communication can alleviate emotional stress and relationship conflict, (7) nurse leaders can inspire and motivate new NMs by role‐modeling, and (8) there is a need for more studies that assess the impact of the training program on the effectiveness of the NM role.

### 4.2. Implications for Future Research

This study’s findings highlight the importance of implementing a training program to prepare the new leader in nursing. Therefore, there is a need to implement experimental studies that include certain training on leadership or manager programs and test their potential to improve the skills of the new manager. In addition, there is a need to conduct more qualitative studies to help clarify the transition experience from novice to experienced NM role.

### 4.3. The Study’s Strengths and Limitations

The NMs provided responses about their experience as NMs. However, we cannot generalize this study to a large population.

## 5. Conclusion

Participants in this study provided insights regarding the transition into the role of nursing managers, as represented by positive and negative experiences. Positive experiences were illustrated by the willingness to wear the administrative role, support and guidance received either from the manager or from the subordinate, and the presence of networking and empowerment to be effective as a new NM. Negative experiences included a lack of preparedness, a lack of autonomy, and a sense of frustration due to insufficient support. Therefore, nursing administrators can provide support, resources, and support programs to new NMs and mentorship when transitioning to a managerial position.

## Conflicts of Interest

The authors declare no conflicts of interest.

## Funding

No funding was received for this manuscript.

## Data Availability

The data that support the findings of this study are available on request from the corresponding author. The data are not publicly available due to privacy or ethical restrictions.
